# Reply to Martínez-Ortíz et al.: High moment of inertia of foxes inhibits their rotation

**DOI:** 10.1073/pnas.2411226121

**Published:** 2024-08-01

**Authors:** Jisoo Yuk, Sunghwan Jung

**Affiliations:** ^a^Department of Biological and Environmental Engineering, Cornell University, Ithaca, NY 14853

In Martínez-Ortíz’s experiment (1), they demonstrated that when a fox skull was dropped onto an expanded polystyrene (EPS) bed, the asymmetry of the skull caused it to rotate. The rotation of an asymmetric object penetrating an interface has been a well-studied and observed phenomenon (2–4). They argued that our experiment oversimplified the problem by using a slider to vertically drop a fox skull equipped with a load cell.

**Fig. 1.**
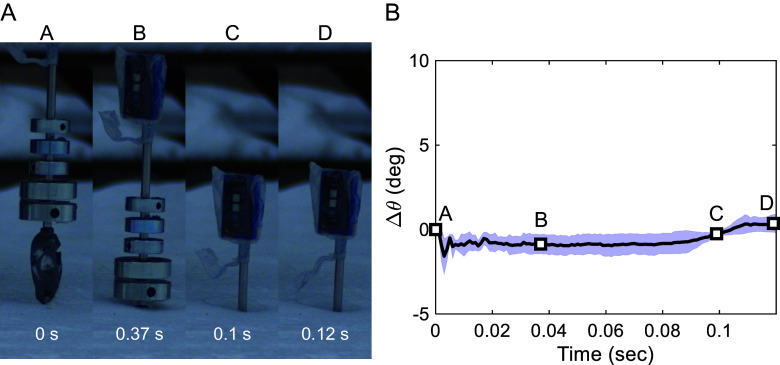
(*A*) Image sequence and (*B*) angular change of the Pale fox (*V. pallida*) skull during free fall on snow in the field test.

Here, we contend that our vertical drop experiment is sufficient to explain the initial impact force on the fox skull. Although not included in the published paper (5), we performed an additional experiment where a Pale fox (*Vulpes pallida*) skull was freely dropped onto a snow-covered field at a speed of 3 m/s (Fig. 1*A*). We attached a weight of 2.9 kg (6), corresponding to the typical weight range of a Pale fox, to the skull to simulate the actual force experienced during a fox’s snow diving. Using a high-speed camera, we recorded the free fall and examined the in-plane angular changes. Despite using a Pale fox skull instead of an Arctic fox skull, we observed that the experimental trends were similar due to the shared pointed skull morphology within the *Vulpes* genus (5).

Our experiment showed minimal angular change from the initial contact (sequence A) to complete burial in the snow (sequence B), within 1.6 degrees (Fig. 1*B*). Poststopping (sequence C to D), the angular change was within 1 degree. Thus, we believe the influence of rotation can be considered negligible. In Martínez-Ortíz’s experiment, it also appeared that there is minimal angular difference up to approximately 0.16 s, the moment the skull becomes submerged. However, they did not consider the actual mass of the animal on the back side of the skull.

The differences in rotational response between our experiment and Martínez-Ortíz’s are likely attributed to significant variations in the weight attached and the medium used. To emulate actual fox diving in our experiment, we matched the typical weight of the Pale fox and placed the center of the weight close to the chest location of the fox. When a skull enters a medium, distributed lateral forces could generate a torque, causing rotation. At this point, if an object has a larger moment of inertia, it will have smaller angular acceleration, thereby reducing the rotational effect. Similarly, adding the average weight of a fox to the skull increases its moment of inertia, minimizing rotation. Additionally, EPS and snow have different medium properties. Snow is a compressible granular medium (7, 8), and it also differs from EPS in terms of density, particle size, and roughness. Since the characteristics of the medium are key factors influencing the impact dynamics (9, 10), they could have affected the experimental results. Thus, under these conditions, particularly focusing on the initial impact, our vertical free fall experiment is deemed sufficiently valid. However, the effects of the front paws, the soft tissues, or an open jaw were not addressed in our experiment, indicating a need for further investigation.
